# Microstructural modifications in bitumens rejuvenated by oil from pyrolysis of waste tires

**DOI:** 10.3389/fchem.2024.1512905

**Published:** 2025-01-30

**Authors:** Michela Alfe, Valentina Gargiulo, Giovanna Ruoppolo, Francesco Cammarota, Pietro Calandra, Cesare Oliviero Rossi, Valeria Loise, Michele Porto, Roberto Di Capua, Paolino Caputo

**Affiliations:** ^1^ CNR-STEMS, Institute of Sciences and Technologies for Sustainable Energy and Mobility, National Research Council, Napoli, Italy; ^2^ CNR-ISMN, Institute for the Study of Nanostructured Materials, National Research Council, Montelibretti, Italy; ^3^ Department of Chemistry and Chemical Technologies, University of Calabria, Rende, Italy; ^4^ Department of Physics “E. Pancini” University of Naples Federico II, and CNR-SPIN, Naples, Italy

**Keywords:** bitumen, pyrolysis oil, rheology, waste tires, microscopy

## Abstract

**Introduction:**

Bitumen is the viscoelastic fluid binding the crushed stones and mineral aggregates in the asphalt material used to pave roads around the world. During the paving procedure, the volatile compounds are lost and oxidization occurs with variation of the mechanical characteristics (aging); thus, the material becomes rigid and brittle over time and may need replacement. Instead of being landfilled, aged asphalts can be reused in new pavements after pretreatment with specific additives to restore their original properties.

**Methods:**

By considering conscious utilization of natural resources, we propose using the condensable fraction (oil) obtained from the pyrolysis of waste tires (WTs) as the agent to rejuvenate aged bitumen. The pyrolysis oil from WTs was produced and characterized using elemental analysis, gas chromatography coupled with mass spectrometry (GCMS), and thermogravimetry. Bitumen was aged by the rolling thin-film oven test procedure and blended with the WT pyrolysis oil at three different concentrations (1%, 3%, and 6% w/w) to evaluate the rheological behaviors. The blends were also investigated using atomic force microscopy, and the asphaltenic fraction was assessed via optical microscopy.

**Results and discussion:**

All the data consistently indicate that oil addition not only reduces the viscosity of bitumen and restores it to values close to the original unaged bitumen but also changes the intermolecular structure to recover the self-assembly pattern typical of the unaged sample. The physicochemical mechanisms of this phenomenon are proposed in light of the oil characteristics. Hence, it is concluded that the pyrolysis oil from WTs can be used to rejuvenate asphalts, which can then be used in reclaimed asphalt pavement technology. The impacts of our findings are expected to be extensive because bitumens are globally used for paving roads. In addition, since the proposed method couples/fuses urban waste treatment with asphalt maintenance processes, two types of wastes (oil from pyrolysis of WTs and aged bitumens) can be simultaneously recovered and reused to produce new and performing asphalts.

## 1 Introduction

Bitumen is a byproduct of crude oil refining and is consumed in significant quantities annually in the construction and maintenance of roads (Penki and Rout, 2021). Bitumen is a non-renewable material, so there is an urgent need to find renewable and sustainable alternatives to petroleum-derived bitumen to improve the resilience, sustainability, and circularity of the roadwork industry. Among the various possibilities, the use of biobased materials is one of the most promising alternatives (Mahssin et al., 2021; Zhang et al., 2022, 2024). Another possibility is to attempt to restore the properties of bitumen in aged asphalts, thereby reducing the need to replace aged asphalts with new ones. The loss of volatile compounds during the paving process as well as exposure to ultraviolet radiation, light, moisture, and oxidation can cause changes in the mechanical and physicochemical properties of asphalt (aging); thus, the material becomes brittle, stiff, and prone to cracking, thereby requiring replacement (Ameri et al., 2023). Instead of being landfilled, aged asphalts can be reused if they can be treated with specific additives to restore their pristine properties. These additives are expected to reduce the viscosity of aged bitumen and sometimes induce structural changes to partially restore the original intermolecular structure and self-assembly pattern of the material. In such cases, these additives are called “rejuvenators,” as opposed to other types of substances (additives, softening agents, or fluxing agents) that simply reduce the viscosity without causing any internal structural changes (Loise et al., 2019; Li et al., 2021; Schwettmann et al., 2023; Zhang et al., 2024). The rejuvenation mechanism is based on the penetration, diffusion, and fusion of the rejuvenator molecules in the old bitumen, thereby modifying its thermomechanical properties to be closer to those of new bitumen. Various rejuvenators have been identified, which are both bio-sourced and not bio-sourced. Rejuvenators can be divided into three types according to their sources: petroleum-based, coal-tar-based, and biobased rejuvenators. Used cooking oil and pyrolysis oil have been recognized as biobased rejuvenators for aged bitumen.

Pyrolysis oil is a complex mixture of water as well as soluble and insoluble organic compounds with a wide range of molecular weights. Examples of molecules that comprise pyrolysis oil are aromatic hydrocarbons like light species (naphthalene, indene, and toluene), ketones, aldehydes, small organic acids, and some light-oxygenated aromatic compounds (phenol, catechol, and lignols). The primary elemental components of pyrolysis oil are carbon (C), hydrogen (H), oxygen (O), and nitrogen (N). Depending on the feedstock and operating conditions, the composition and water content of bio-oils can vary (Zhang et al., 2022). It has been reported that pyrolysis oils can effectively soften aged bitumen by reducing its stiffness and increasing its flexibility, thereby improving the low-temperature performance and fatigue resistance of the material while maintaining a reasonably efficient high-temperature performance. Liu et al. (2023) investigated the possibility of using bio-oils from the pyrolysis of corncob and birch bark as biobased rejuvenators to regenerate aged asphalt binders; they found that light molecular compounds are the major contributors to the rejuvenation of aged asphalt binders. Since the earliest attempts to use pyrolysis oils as asphalt rejuvenators, it has been clear that the aromatic components of these oils play crucial roles: aromatic molecules tend to interact well with aged bitumen and asphalt components, as their chemical compositions and physicochemical properties are similar to petroleum-derived rejuvenators. Li et al. (2021) investigated the use oils from the pyrolysis of wood and waste tire (WT) rubber as well as co-pyrolysis of both as aged bitumen rejuvenators with the aim of highlighting the roles of the compositions of such bio-oils on the rejuvenation process; they concluded that for a pyrolysis oil to be effective as a rejuvenator, it must contain a balanced combination of aromatics and ketones. Pyrolysis oil from scrap tires was also tested as a possible asphalt rejuvenator by El-Ashwah and Abdelrahman (2024), and they found that 12% scrap tire pyrolysis oil was the most effective blend proportion for improving fatigue and low-temperature cracking resistance that allowed improved interactions between the virgin binder and recycled asphalt materials.

In the present work, we further investigated pyrolysis byproducts as possible ingredients in asphalt preparations (Caputo et al., 2022a; Gargiulo et al., 2023) by exploring the molecular aspects involved in the use of pyrolysis oil from the pyrolysis of WTs as bitumen rejuvenators. Accordingly, the pyrolysis oil was characterized and blended at low percentages (1%, 3%, and 6% w/w) with aged bitumen. The results reported herein encourage reliable use of the pyrolysis oil from WTs in reclaimed asphalt pavement (RAP) technology for asphalt maintenance processes while also providing a viable solution to the treatment of WTs.

## 2 Experimental procedures

### 2.1 Materials

The bitumen used in this study was produced in Saudi Arabia and supplied by Lo Prete (Italy). Its penetration grade was 50/70 (low-penetration-grade bitumen, hereafter denoted as LP) as measured by the usual standardized procedure (ASTM D946) (Petrauskas and Ullah, 2015); in this procedure, a standard needle is loaded with a weight of 100 g, and the length traveled into the bitumen specimen is measured with a resolution of tenths of a millimeter over a known time at a fixed temperature. The characteristics of the bitumen used herein have been reported in previous works (Loise et al., 2021; Caputo et al., 2022b). The concentrations (w/w %) of the four main bitumen components (saturates, aromatics, resins, and asphaltenes) evaluated by the S.A.R.A. method were found to be 3.8, 51.3, 21.5, and 23.4, respectively. All chemicals used were of ACS grade purchased from Merck KGaA (Darmstadt, Germany) and used as received.

### 2.2 Pyrolysis oil production and characterization

The pyrolysis oil was derived from scrapped WTs. Here, WT powder having an average particle size of 0.8 mm and mainly composed of C (82.3% w/w) and H (6.1% w/w) (Supplementary Table S1) was pyrolyzed up to 550°C under nitrogen flow in a lab-scale experimental apparatus described previously (Gargiulo et al., 2023). The condensable species (hereafter referred to as pyrolysis oil) evolved during the pyrolysis process were conveyed to the condensation system comprising two refrigerated flasks (−4°C) for collection. The pyrolysis test was repeated twice, and the amount of feedstock involved was approximately 350 g for each test.

The C, H, and N contents of the feedstock and the pyrolysis products, both condensable species and char (i.e., solid product of the pyrolysis process), were determined by ultimate analysis in accordance with the ASTM D3176-15 standard protocol using a LECO 628 analyzer calibrated with EDTA. The measurements were repeated three times. The water concentration in the oil was determined via Karl–Fisher titration (Mettler Toledo V20 Karl Fischer volumetric titrator) based on three measurements. The thermal behaviors of the feedstock and pyrolysis products were analyzed via thermogravimetric analyses on a STA6000 Perkin-Elmer thermobalance under both inert (N_2_ at 40 mL/min) and oxidizing (air at 40 mL/min) atmospheres. Each sample (2–20 mg) was loaded in an alumina crucible and heated from 30°C to 800°C at the rate of 10°C/min. The alumina crucible was previously preheated to 920°C in air to guarantee accurate determination of the solid residue.

The pyrolysis oil was analyzed by gas chromatography mass spectrometry (GC-MS) without further derivatization. The samples were dissolved in acetone (dilution 1:10 by volume), and 1 μL of the solution was analyzed on the Agilent GC-MS instrument (7890A/5972C) equipped with an Agilent DB-624 capillary column (30 m × 0.25 mm i.d., 1.40 μm film thickness). Helium was used as the carrier gas (1.0 mL/min). The mass spectrometer was operated in the electron ionization mode, and the m/z range of 30–400 was scanned. The oven temperature was programmed as follows: the starting temperature was set at 45°C and maintained for 4 min; then, the temperature was increased to 235°C at a ramp rate of 3°C/min and maintained for 60 min (total runtime of 127 min).

### 2.3 Bitumen blend preparation

The pyrolysis oil was added at the desired content (1%, 3%, and 6% w/w) to fully flowing hot bitumen (150°C ± 10°C) to prepare the oil-modified material. Then, a mechanical stirrer (IKA RW20, Königswinter, Germany) was used at 500–700 rpm (30 min) for blend homogenization. Our experience suggests that these conditions can best ensure homogeneity of the samples: the sample homogenization is not effective at lower rpm values, and the bitumen may be oxidized above 700 rpm owing to the increased bitumen contact with air and related kinetics. We consider the proposed method as a standard operation that is coherent with procedures reported by other authors (Shaffie et al., 2018). After mixing, the final bituminous sample was poured into a small sealed can and stored in a dark chamber at 25°C. Care was taken to always maintain the same operating conditions to ensure that the results were comparable and to avoid variations due to different annealing times (Rossi et al., 2017).

### 2.4 Aging tests

To simulate sample aging, the rolling thin-film oven test (RTFOT) procedure was adopted according to ASTM D2872-04 guidelines. The apparatus consisted of an internal double-wall furnace in which hot air was circulated at the test temperature of 163°C driven by an internal fan. Eight specially designed glass specimen bottles were mounted horizontally in a carousel within the oven. The test involved subjecting a thin layer of bitumen (∼1.25 mm) to a jet of hot air for 75 or 225 min. Each modified bitumen sample along with a reference binder free of additives was divided into two aliquots, of which one was subjected to the artificial aging process. The aging time lasted 225 min instead of the commonly adopted 75 min to simulate a prolonged aging period of approximately 10–12 years, which is comparable to the typical lifecycle of asphalt.

### 2.5 Rheological tests

The mechanical properties of the samples were investigated via dynamic shear rheological (DSR) tests, in which the complex shear modulus (Hunter, 2015) given by G* = G′ + *i*G″ was measured as a function of temperature; the temperature was controlled using a Peltier element, with an uncertainty of ±0.1°C and ramp temperature of 1°C/min. A dynamic stress-controlled rheometer (SR5000, Rheometric Scientific, Piscataway, NJ, United States) equipped with a parallel plate geometry (gap of 2 mm in agreement with literature (Lu et al., 2021) and diameter of 25 mm) was used for this purpose. The small-amplitude oscillatory shear regime (Remišová et al., 2018) was maintained after the preliminary stress–sweep tests to guarantee linear viscoelastic conditions for all measurements with a frequency of 1 Hz. The real and imaginary parts define the in-phase (storage and measure of reversible elastic energy) and out-of-phase (loss and irreversible viscous dissipation of mechanical energy) moduli, respectively (Barnes et al., 1989). The bitumen sample weighing approximately 2 g was placed on the plate of the rheometer and heated to 100°C until it melted; then, the rheometer tool was lowered to the desired gap. The experiments were conducted once the test temperature was reached.

### 2.6 Bitumen and blend deasphaltenization procedure

The asphaltenic fractions were extracted from the samples according to ASTM D6560 as reported in an earlier work (ASTM, 2000; Rossi et al., 2018). The pentane-insoluble asphaltenic fractions were recovered under vacuum filtration, washed thoroughly, and dried in an oven at 80°C for 3 h.

### 2.7 Optical microscopy analysis

Optical microscopy analysis was carried out using a Leica DMLP polarizing microscope equipped with a Leica DFC280 camera and a CalCTec (Italy) heating stage. Double microscope glass slides were used (sandwich model) to host the samples. The temperature was increased at the rate of 5°C/min starting from 120°C, and photographs were captured after thermal stabilization for 1 min at each 5°C increment. It is noted that the heating rate of 5°C/min adopted in the present work is higher than that typically used to measure the melting points of organic solids. This is intended to avoid eventual changes during the measurements owing to the reactive nature of the samples (Caputo et al., 2025). Interesting considerations related to this can be found in literature (Gray et al., 2004).

### 2.8 Atomic force microscopy (AFM) analysis

AFM images were acquired using an XE100 Park instrument operating in the non-contact mode (amplitude modulation, silicon nitride cantilever from Nanosensor) at room temperature under ambient conditions. A conical tip of 10 nm nominal end radius was used, and the resonance frequency was set to 150 kHz. Samples for the AFM investigations were prepared by placing a palette containing a small amount of bitumen on the sample holder and maintaining this in an oven at a fixed temperature of 100°C for 10 min. Under these conditions, the bitumen becomes fluid and uniformly covers the entire sample. Thereafter, the sample was gently cooled by opening the door of the oven, and the sample returned to room temperature in approximately 30 min. This simple procedure allowed preparation of a bituminous sample with a smooth surface for AFM analysis. Precautions were maintained to ensure that the same operational conditions were applied to all the samples for safe comparisons.

## 3 Results and discussion

### 3.1 Pyrolysis oil composition and thermal properties

The pyrolysis oil derived from WTs was characterized by a carbon content of 75.2 wt.%, hydrogen content of 9.4 wt.%, and very low nitrogen content of 0.2 wt.%. The water content determined by Karl–Fisher titration was below 1 wt.%. As expected, the H/C atomic ratio of the pyrolysis oil is higher than those of the starting feedstock and char (Supplementary Tables S1, S2) since most of the aliphatic and aromatic species evolutions during the pyrolysis test are collected in the oil. Interestingly, the S content (accounting for 2.3 wt.% in the WTs) was totally segregated in the WT char (3.2 wt.%) as it was not detectable in the pyrolysis oil.

**FIGURE 1:**
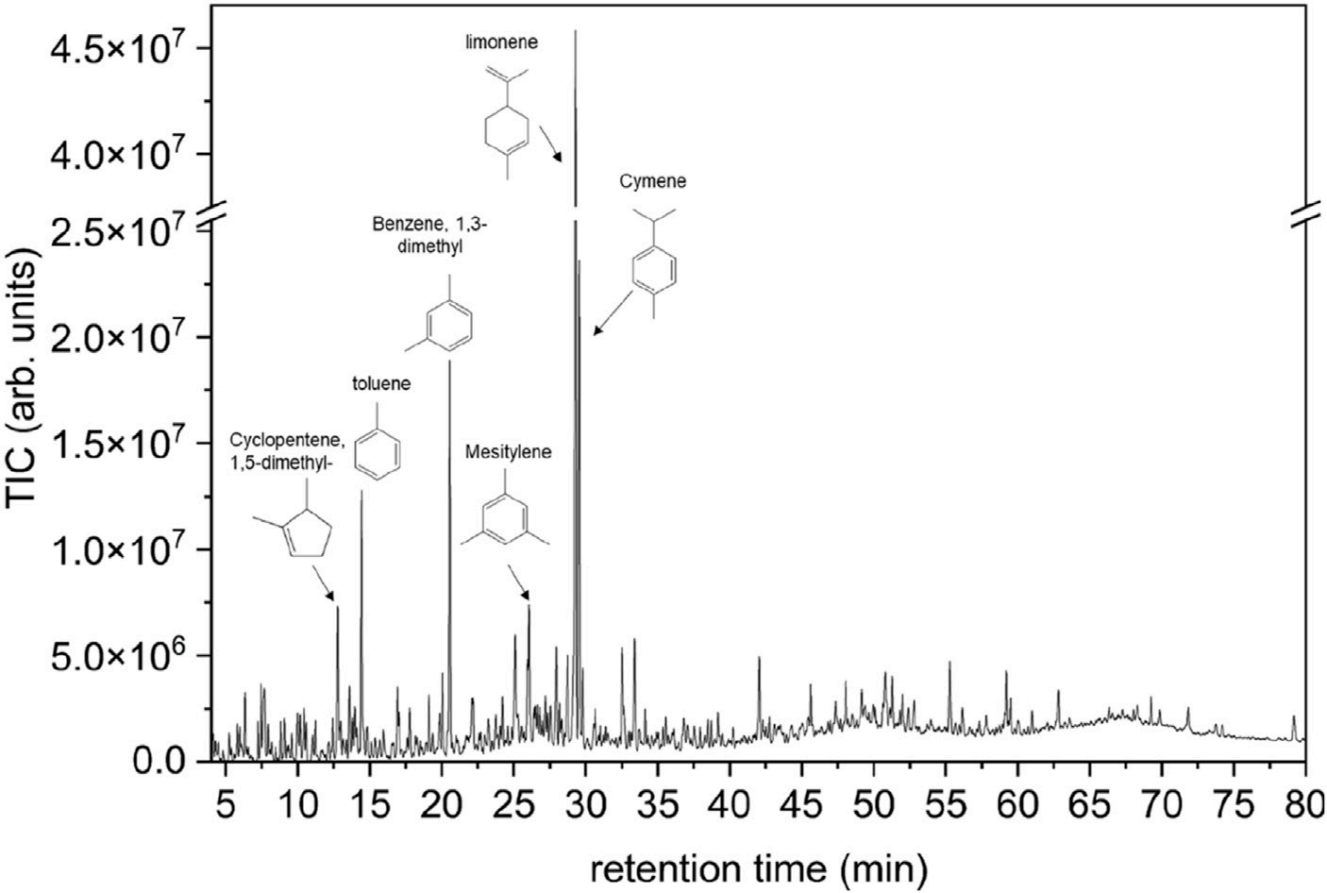
Total ion chromatogram (TIC) of the pyrolysis oil from waste tires (WTs) showing the most abundant components.

The chromatogram obtained from the GC-MS analysis of the pyrolysis oil is shown in Figure 1, and the main species identified by comparisons with available instrumental libraries are listed in Supplementary Table S3. The WT pyrolysis oil is a mixture of aromatic compounds; here, limonene (1-methyl-4-(1-methylethenyl)-cyclohexene) is the most abundant compound, followed by some alkyl-benzene derivatives such as mesitylene and cymene, naphthalene derivatives, as well as traces of sulfur-containing compounds (benzothiazoles). The formation of aromatic derivatives of benzene, toluene, and xylene (BTX) during WT pyrolysis is usually ascribed to secondary limonene conversion (Campuzano et al., 2020; Januszewicz et al., 2020; Ye et al., 2022; Zhang et al., 2022). The composition of the WT pyrolysis oil is in line with the results reported in literature (Alvarez et al., 2017; Campuzano et al., 2020; Januszewicz et al., 2020; Zhang et al., 2021).

**FIGURE 2:**
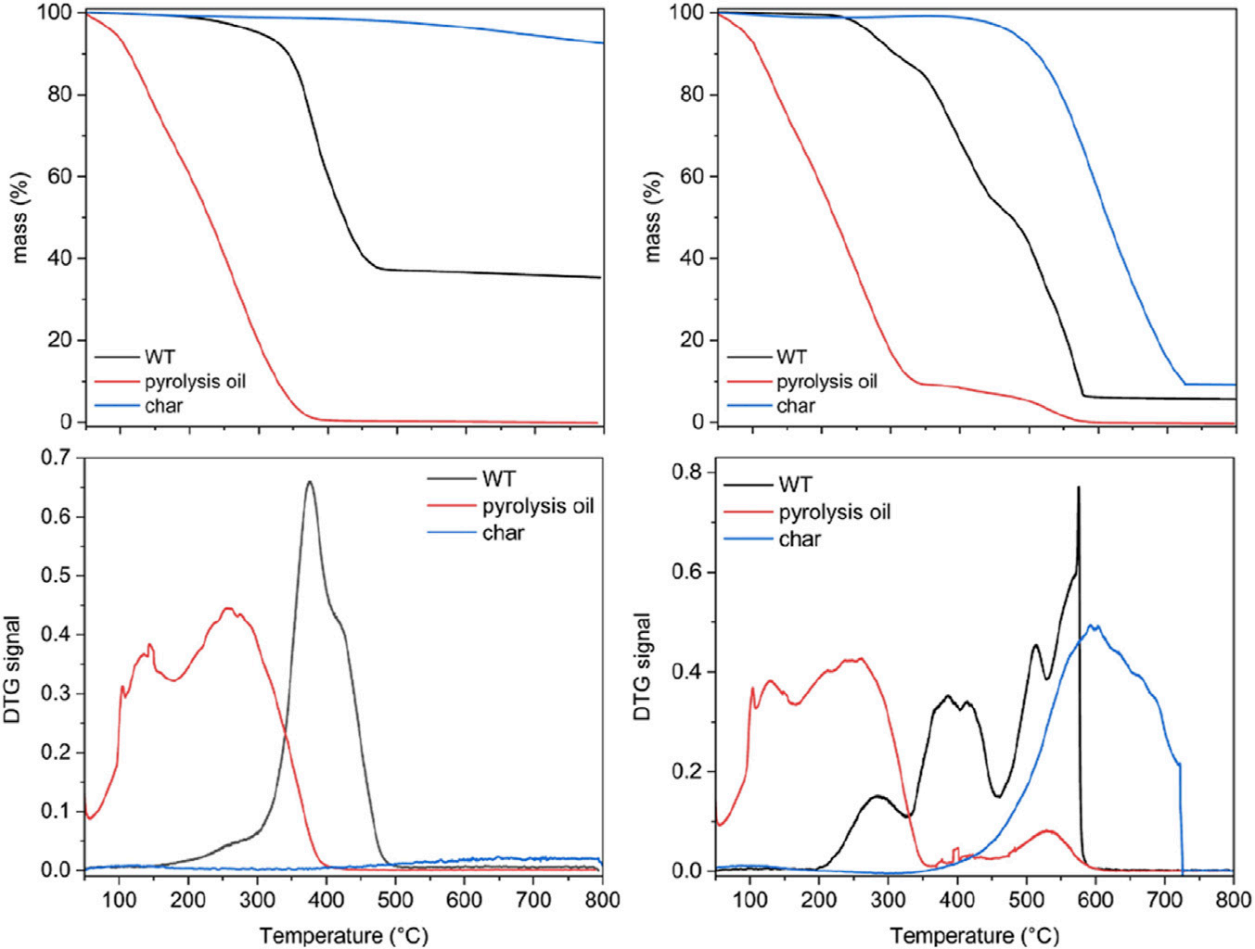
Thermal behaviors of the WTs and pyrolysis products (oil and char) under inert (N_2_ at 40 mL/min) and oxidizing (air at 40 mL/min) atmospheres. Results obtained under inert atmosphere (N2 at 40 mL/min are reported in the panels on the left, while results obtained under oxidizing atmoshpere (air at 40 mL/min) are reported in the panels on the right.

The thermal behaviors of the WT pyrolysis oil are quite different from those of the starting feedstock and char under both inert and oxidizing atmospheres (Figure 2) as it is a mixture of hydrogenated low-molecular-weight compounds. Under inert atmospheres, the pyrolysis oil volatilizes completely before reaching 350°C without the formation of any detectable residues; under an oxidizing atmosphere, after the main thermal event peaks before 320°C corresponding to a loss of approximately 90 wt.%, another thermal event that peaks at approximately 500°C is detected, which is attributable to the combustion of heavy residues. The thermal behaviors detected under both inert and oxidizing atmospheres are in agreement with the findings of previously reported literature on WT pyrolysis oil (Gani et al., 2022).

### 3.2 Rheological tests

The rheological properties were measured through the time cure test, in which G′ and G″ are monitored during a temperature ramp (at a constant heating rate of 1°C/min) at a fixed frequency (1 Hz). Upon heating, the samples become progressively softer with a monotonous decrease in G′. In this situation, when the molecular relaxation rate is sufficiently high for the system to accommodate mechanical distortions/perturbations, the bitumen can be considered to almost be a Newtonian fluid and G′→0. This is a real gel-to-sol transition, and the corresponding temperature (T*) can be considered to represent the rigidity of the sample. The values of T* are reported in Table 1 for various samples. Some conclusions can be drawn from these observations. First, the transition temperature is clearly affected by the aging process since oxidation processes enabled mainly by atmospheric oxygen cause the formation of more polar molecules. In turn, this favors the aggregation of molecules, which obviously increases the overall rigidity. Another cause for this increase in rigidity is the loss of the more volatile components through evaporation. As a consequence of these phenomena, G′ increases from 64.6°C to 79.5°C.

**TABLE 1:** Rheological parameters of the samples.

Sample	T*(±0.1) (°C)	G′ @ 50°C (±3%) (Pa)	Ea (kJ/mol)
LP	64.6	2,720	136.1 ± 0.2
LP aged for 75 min	69	5,335	123.3 ± 0.2
LP aged for 225 min	79.5	28,948	130.4 ± 0.3
LP aged + 1% oil	77.7	22,770	134 ± 2
LP aged + 3% oil	75.8	18,488	129 ± 1
LP aged + 6% oil	68.7	4,008	127.7 ± 0.3

The progressive addition of the pyrolysis oil to the aged bitumen gradually lowers the transition temperature. The addition of oil at 6% w/w lowers the T* value by approximately 11°C. The pyrolysis oil therefore acts as a partial regenerator of the original rigidity of bitumen. It is important to note that molecular self-assembly in bitumens is a complicated and complex phenomenon involving the formation of asphaltene clusters that are hierarchically assembled at various length scales (Calandra et al., 2018). It would be interesting to understand if this effect is a result of a viscosity reduction with no change to the intermolecular self-assembly (mere fluxing effect) or an exertion of an effect on the molecular self-assembly (rejuvenation). In the latter case, the oil molecules would add new intermolecular interactions that compete with the asphaltene–asphaltene interactions to disaggregate the overall oxidized asphaltene-based intermolecular network, endowing more fluidity to the system. From the rheological data, it is difficult to understand this structural aspect, so a specific technique like AFM is necessary (see below). However, the origin of the observed rheological effect can be ascribed to the peculiar chemical composition of the oil; despite the very low N content and traces of S atoms (not detectable by elemental analysis), which would theoretically help in establishing specific interactions with the asphaltene molecules, the oil retains most of the aliphatic and aromatic species that evolved during the pyrolysis test, thereby enriching the maltene phase.

**FIGURE 3:**
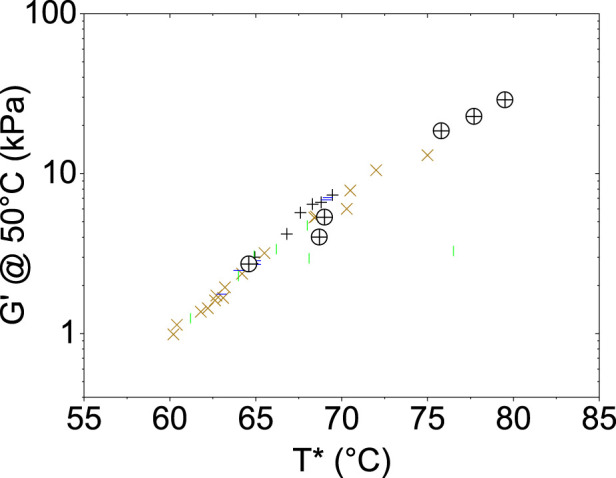
Correlation between G′@50°C and T* in bitumens reinforced with polysaccharides (Porto et al., 2019) (brown crosses), inorganic fine particles (Calandra et al., 2022) (horizontal green bars), char from pyrolysis (black crosses), and cellulose (Caputo et al., 2023) (vertical green bars), as compared to the data from the present work (crossed circles).

Another interesting and independent indicator of the rigidity of the system is the value of G′ under working conditions, i.e., usually 50°C. This has often been considered as a useful quantity to assess the mechanical properties of bituminous materials (Somé, 2016). In agreement with literature, this quantity will be indicated as G′@50°C hereafter, and its values are reported in Table 1 alongside T* values for better comparison. As seen from the table, G′@50°C and T* are well-correlated even though they are two independent parameters. This reinforces the findings derived from the analysis of T* and particularly the regenerative effects of the bio-oil. Furthermore, if these data are combined with those from literature with reference to other bituminous samples (as per the correlation plot for the huge comparison dataset in Figure 3), it can be seen that all the datasets are in agreement. Interestingly, this suggests a universal behavior.

Further analyses can be performed by considering the temperature dependences of both G′ and G″. In fact, from G′ and G″, the viscosity (η) can be defined as (Macosko et al., 1994) by Equation 1:

$$\eta =\frac{G}{\omega }=\frac{\sqrt{G^{\prime 2}+G^{\prime \prime 2}}}{\omega }.$$ (1)

This quantity is related to the total amount of energy absorbed by the system under oscillatory shear conditions. Usually, this energy is observed as resistance to flow, so it is well-suited for use in bituminous systems. The Arrhenius plot reporting the temperature dependence of η shows an obvious linear trend in the temperature interval excluding the gel-to-sol transition range (25°C–55°C). Hence, the two-well potential model can be used to obtain Equation 2:

$$\ln\eta *\left(T\right)=\ln As+\frac{Ea}{R}\cdot \frac{1}{T},$$ (2)

where R is the gas constant (8.314 J/K·mol), and the apparent activation energy *Ea* of the flowing process can be derived. The derived *Ea* values are also reported in Table 1 for comparison with T* and G′@50°C. Some interesting observations are due here: the *Ea* values are in the range 120–130 kJ/mol and are consistent with the values observed for other bituminous materials (Calandra et al., 2018). Moreover, the *Ea* values are not correlated with the T* values, which is again consistent with previous observations (Porto et al., 2019), suggesting that the mechanism of flowing is presumably uncoupled with that responsible for the gel-to-sol transition.

**FIGURE 4:**
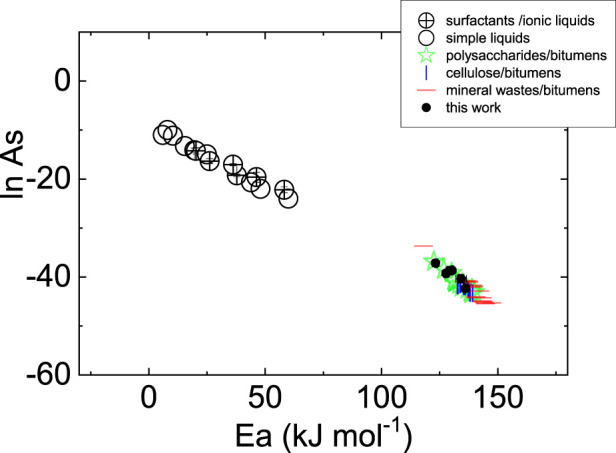
Correlations between the Arrhenius parameters for a wide variety of materials (shown in legend).

Interestingly, *Ea* and the preexponential factor (ln *As*) are correlated (Figure 4). Furthermore, this peculiarity has been observed in simple liquids (Messaâdi et al., 2015) as of 2015, even if simple liquids are quite different from the materials under investigation in the present work. To show that this behavior is universal, Figure 4 reports data from other bituminous samples and surfactant-based liquid mixtures as well as ionic liquids. The variety of samples that conform to the correlation seems to be striking and leads to a mere applicative consequence: the temperature behavior of viscosity can be described/predicted even if only one Arrhenius parameter is known since the other can be derived thereof, which deserves attention. However, from a basic science perspective, more work is needed to support the theoretical investigations to understand the molecule-based principles beyond this correlation given the absence of any theoretical precedent. In this regard, the data presented in this work are expected to help with data collection for this purpose.

The hypothesis that the pyrolysis oil can be dispersed at the molecular level is consistent with its eventual rejuvenating effect, where the pyrolysis oil restores not only the ductility to that of pristine bitumen like any other fluxing agent but also the molecular structure. The changes in *Ea* for these samples (data reported in Table 1) cannot be easily rationalized, where a certain decrease in *Ea* with the oil content is observed. From a microscopic point of view, this change may be interpreted in terms of the molecular mechanism responsible for shear. As concisely described by Bryan et al. (2005), “*For any one molecule to move, other surrounding molecules must first give way and move into vacant lattice sites or “holes” to create a space for the molecule to enter.*” Accordingly, the effect of decreasing *Ea* would be the consequence of establishment of loose oil–bitumen interactions at the molecular level, consistent with the experimental observations reported previously. In any case, there is no easy interpretation for the changes in *Ea*, so it is possible to claim an interplay among different mechanisms from the microscopic point of view (like aging effects, presence of bio-oil, and complex molecular dynamics occurring in bituminous materials).

### 3.3 Optical microscopy

**FIGURE 5:**
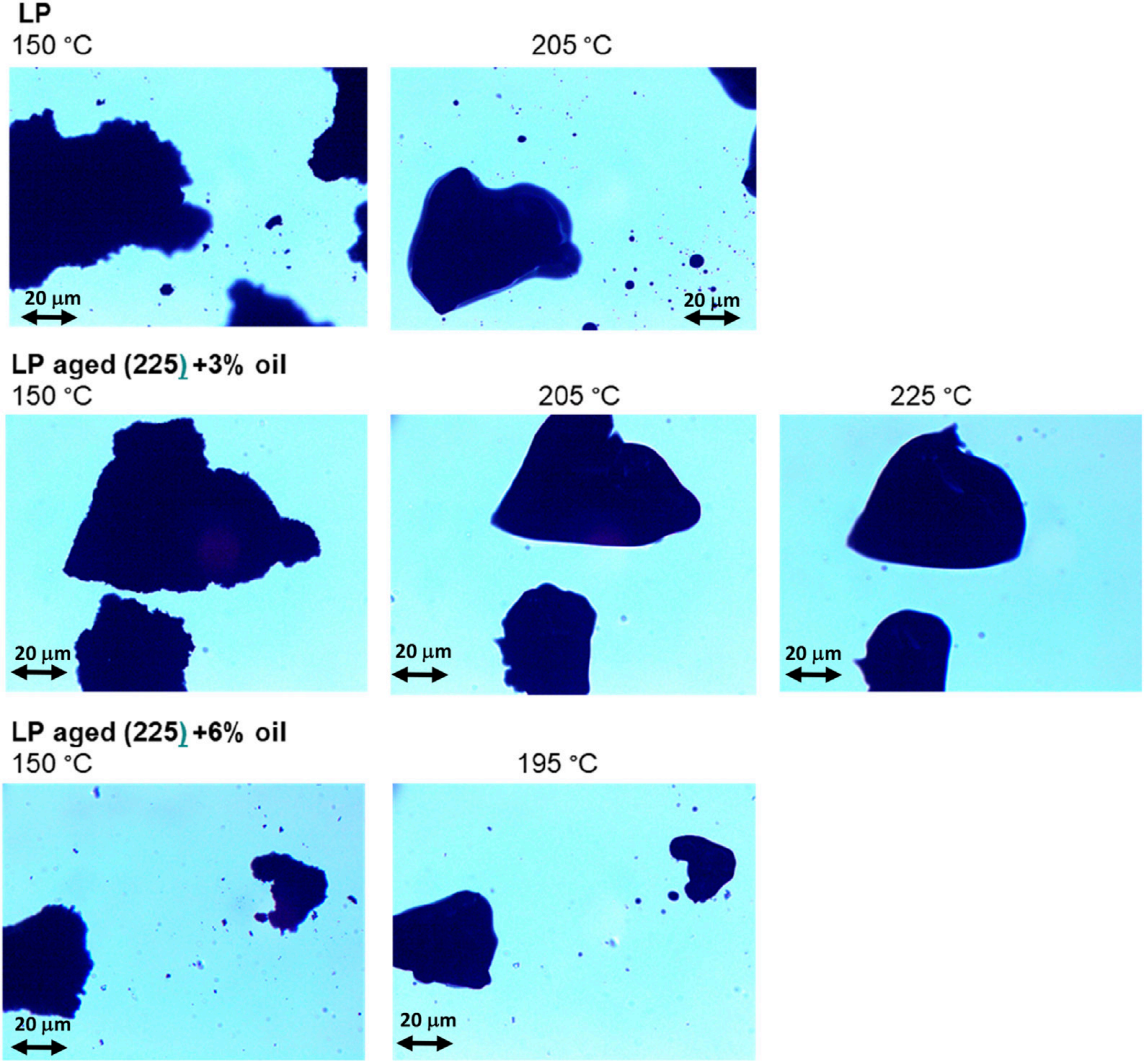
Optical microphotographs of the asphaltene clusters subjected to heating.

Table 2 presents the melting points of the asphaltene fractions of the samples. The images of the asphaltenic sample fractions subjected to increasing temperature conditions were acquired at a fixed rate by optical microscopy to determine the melting points. This approach adopts a higher heating rate (5°C/min) than that typically adopted in conventional methods (heating the sample in a capillary tube) and is helpful in the case of reactive materials like asphaltenes. The images (Figure 5 showing some representative samples) were recorded from the asphaltenic fraction of bitumen after asphaltene separation. The aim here is to measure the melting point of this fraction, so its isolation from the maltenic component rules out optical obstacles from the latter. This method is considered useful in determining the state of the asphaltenic cluster since it reveals the inherent characteristics of the asphaltene fraction that has a pivotal role apart from its mere abundance in the bitumen. In the optical microscopy images, micrometer-sized aggregates can be observed. Some aggregates larger than those usually observed in suspension (Li et al., 2018) are also found since the growth phenomenon can occur due to the progressive increase in nanoparticle concentration during sample preparation (ripening, coalescence, agglomeration, and aggregation during filtration and drying) (Michaelides, 2008).

**TABLE 2:** Melting points of the asphaltene fractions of the samples.

Sample	Melting point of the asphaltene fraction (±0.1) (°C)
LP	185
LP aged for 225 min	205
LP aged (225) + 3% oil	205
LP aged (225) + 6% oil	195

Asphaltene clusters of the pristine bitumen melt at 185°C, but it must be stressed that the temperature reported herein involves a certain interval of a few degrees Celsius in which the solid-to-liquid transition occurs, consistent with previous observations (Zhang et al., 2003). This is attributed to the fact that asphaltenes are not singly defined molecules but rather a class of molecules including a wide variety of chemical species. In addition, their complex aggregation patterns include large-scale asphaltene aggregates that can progressively disperse over a wide temperature range (Tanaka et al., 2003; Thiyagarajan et al., 2003).

The melting point of the asphaltene aggregates is obviously higher than that of pristine bitumen (205°C) owing to the more oxidized state of the asphaltenes, which result in stronger asphaltene–asphaltene interactions. It must be noted that 20°C changes in the melting point after aging are rather large, at least when compared with the findings of a previous paper (Loise et al., 2021) that reported a melting temperature increase of bitumen in the range of 5°C–10°C upon implementation of the same aging process. It is noted that the addition of oil causes a reduction in the melting temperature, at least for oil content >3%, demonstrating an effect on the inner structure of the asphaltene clusters. Hence, a certain rejuvenating effect can be claimed, individuating its origin in the asphaltene–asphaltene interactions constituting their assemblies. Small reductions in the carbonyl and sulfoxide indices were noted by El-Ashwah and Abdelrahman (2024) when WT pyrolysis oil was added to aged bitumen; the decreases in the concentrations of such polar functional groups could actually lower the asphaltene–asphaltene intermolecular interactions and consequently the melting point. However, we are cautious in accepting the conclusion that the decreases in the carbonyl and sulfoxide indices occur upon addition of the pyrolysis oil; in our opinion, we cannot exclude a mere effect of “dilution” of the bitumen signals exerted by oil addition to the sample in the work by El-Ashwah and Abdelrahman (2024), which would lead to the same results. On the other hand, according to the data presented herein, the reduction in asphaltene–asphaltene intermolecular interactions could be due to the prevalent aromatic nature of the bio-oil composed of 1,3 dimethyl benzene, limonene, cymene, and toluene as the major constituents of the pyrolysis oil: aromatic molecules tend to interact well with asphaltene-based clusters of aged bitumen as they have a similar aromatic nature. This chemical consideration can be taken as supplementary information to understand the rejuvenating effects of the pyrolysis oil from WT at the molecular level.

### 3.4 AFM

**FIGURE 6:**
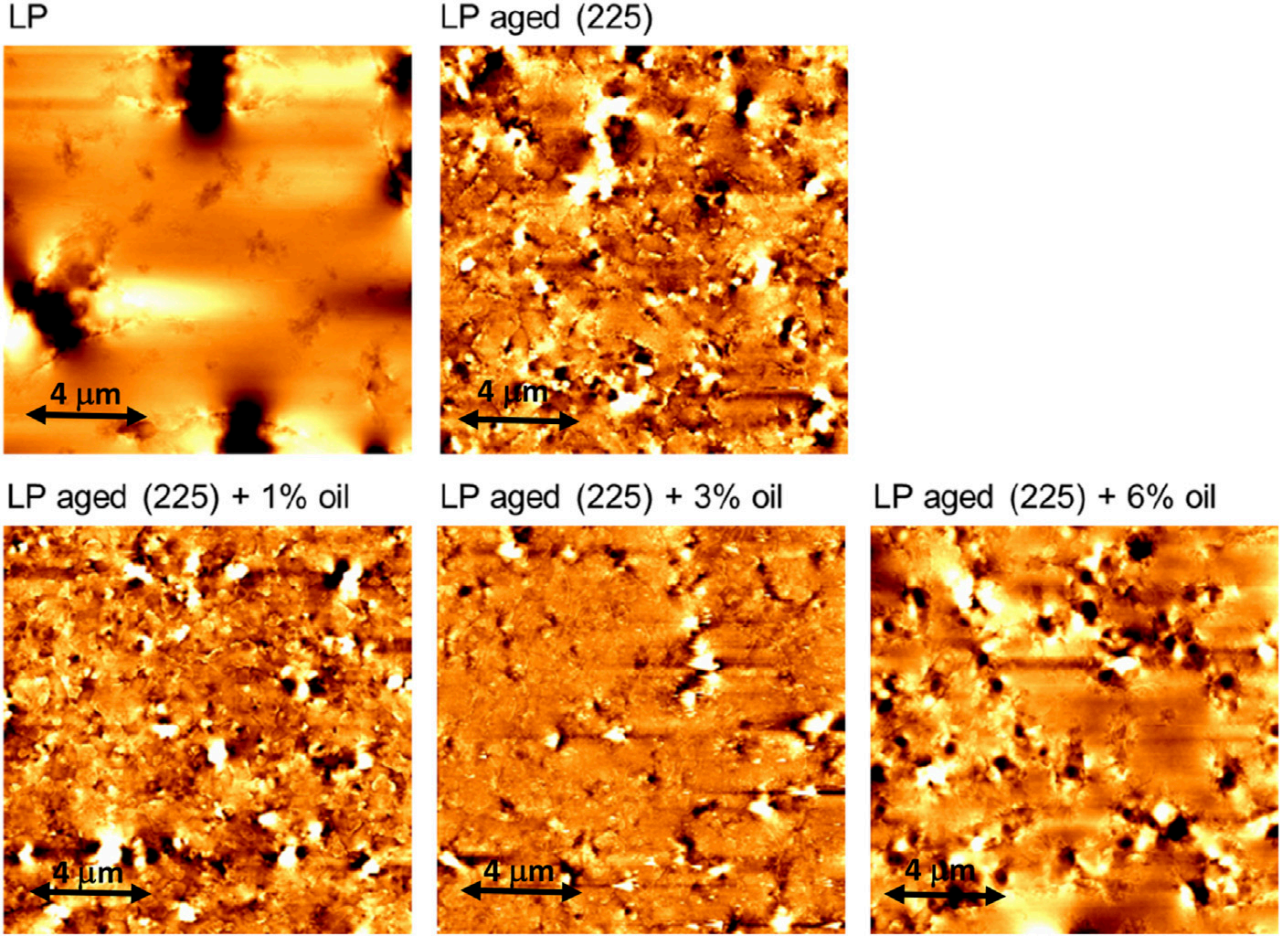
Atomic force microscopy images of the investigated samples.

AFM was used to explore the structures of the bitumens and blends. From the AFM images (Caputo et al., 2025), it is possible to identify different phases as follows: catana phase comprising the so-called bee structures, surrounded by the peri phase, and finally the dominant perpetua phase constituting the matrix. As known from literature (Loise et al., 2021), the aging process reduces the dimensions of the bee structure compared to that of pristine bitumen, and the peri phase appears more interconnected (Figure 6). The AFM images confirm these trends and are in accordance with the rheological results, which indicate a general hardening of the bitumen due to cluster formation and the aged bitumen shows a shift toward higher phase values with respect to those of pristine bitumen. As seen from the figure, the oil has the general effect of slightly reducing the connections between the asphaltenes with respect to the aged bitumen, with only minor effects on the catana phase (i.e., asphaltene cluster size). Thus, a certain rejuvenating effect can be claimed, which is probably attributable to the prevalent aromatic nature of the pyrolysis oil and in agreement with the clues from optical microscopy.

## 4 Conclusion

The oil obtained from the pyrolysis of WTs was successfully tested as a regenerator of aged bitumens to explore the microscopic aspects involved in the rejuvenating effect at the molecular level. Since the oil showed a low content of N atoms and traces of S atoms, which are important to establish oil–asphaltene interactions for rejuvenation, the effects were attributed to the fact that the oil is rich in 1,3 dimethyl benzene, limonene, cymene, and toluene. Aromatic species like these molecules, in fact, tend to interact well with the asphaltene assemblies of aged bitumens owing to their similar chemical nature, thereby restoration of the original asphaltene self-assembly. Consequently, the asphaltene–asphaltene intermolecular interactions are reduced, as observed through the melting point of the asphaltenic fraction, and the asphaltenic bee structures are partially restored to the unaged form, with a less-interconnected network of the catana phases.

## Data Availability

The raw data supporting the conclusions of this article will be made available by the authors without undue reservation.
