# Supplementary material for: Microstructural modifications in bitumens rejuvenated by oil from pyrolysis of waste tires
Source: Front Chem. 2025 Jan 30;12:1512905. doi: 10.3389/fchem.2024.1512905 (PMC11821977; doi:10.3389/fchem.2024.1512905)
Supplement: Supplementary file 1 [file Table1.docx]

**Waste tires as valuable source for reusing waste asphalts**

Michela Alfè^1^, Valentina Gargiulo^1^, Giovanna Ruoppolo^1^, Francesco Cammarota^1^, Roberto Di Capua^2^, P. Calandra^3^, Cesare Oliviero Rossi^4^, Valeria Loise, Michele Porto, Paolino Caputo^4^.

^1^ CNR-STEMS, National Research Council, Institute of Sciences and Technologies for Sustainable Energy and Mobility, 80125 Napoli, Italy

^2^ Department of Physics “E. Pancini” University of Naples Federico II, and CNR-SPIN, Naples, Italy

^3^ CNR-ISMN, National Research Council, Institute for the Study of Nanostructured Materials, Strada provinciale 35 D n.9, 00010 Montelibretti (RM) Italy

^4^ Department of Chemistry and Chemical Technologies, University of Calabria, Via P. Bucci, Cubo 14/D, Rende (CS), 87036, Italy

**Supporting information**

| **Table S1**. WT properties [GArgiulo ColSuA 2023] | |
| --- | --- |
| C (%w/w) | 82.3 |
| H (%w/w) | 6.1 |
| N (%w/w) | 0.1 |
| S (%w/w) | 2.3 |
| Humidity (%w/w) | 0.49 |
| Volatiles (%w/w) | 64.58 |
| Ashes (%w/w) | 6.7 |
| Fixed carbon (%w/w) | 28.23 |
| H/C | 0.89 |

| **Table S2**. Pyrolysis products composition | | | |
| --- | --- | --- | --- |
| WT char [Gargiulo ColSuA 2023] | | WT bio-oil | |
| C (%w/w) | 88.8 | C (%w/w) | 75.2 |
| H (%w/w) | 0.03 | H (%w/w) | 9.4 |
| N (%w/w) | 0.1 | N (%w/w) | 0.2 |
| S (%w/w) | 3.2 | S (%w/w) | n.d. |
| Humidity (%w/w) | 1.6 | water content (%w/w) | < 1 |
| Volatiles (%w/w) | 6.2 |  |  |
| Ashes (%w/w) | 12.5 |  |  |
| Fixed carbon (%w/w) | 79.7 |  |  |
| H/C | 0.004 | H/C | 1.5 |

| **Table S3**. Main species identified in the WT pyrolysis oil GC-MS chromatogram by comparison with a NIST library. | | | |
| --- | --- | --- | --- |
| **RT** | **Area%** | **Library/ID** | **Quality match** |
| 7.614 | 1.27 | 2-Pentene, 4,4-dimethyl-, (E)- | 76 |
|  |  | 3-Hexen-2-one | 70 |
| 12.78 | 1.67 | Cyclopentene, 1,5-dimethyl- | 90 |
|  |  | Cyclobutane, (1-methylethylidene)- | 90 |
|  |  | Cyclopentene, 4,4-dimethyl- | 80 |
| 13.597 | 0.8 | Heptane, 2,2,4,6,6-pentamethyl- | 47 |
|  |  | Pentane, 2,2,4,4-tetramethyl- | 47 |
|  |  | Hexane, 2,2,4-trimethyl- | 47 |
| 14.444 | 2.56 | Toluene | 95 |
| 20.052 | 0.86 | Ethylbenzene | 94 |
| 20.551 | 3.97 | Benzene, 1,3-dimethyl- | 97 |
| 22.118 | 0.95 | o-Xylene | 94 |
| 25.106 | 1.31 | Cyclohexene, 4-ethenyl-1,4-dimethyl- | 93 |
|  |  | D-Limonene | 90 |
| 26.069 | 2.64 | Benzene, 1-ethyl-2-methyl- | 94 |
|  |  | Mesitylene | 87 |
| 27.973 | 1.28 | Benzene, 1,2,4-trimethyl- | 87 |
|  |  | Benzene, 1,2,3-trimethyl- | 60 |
| 28.73 | 1.62 | Cyclohexene, 1-methyl-4-(1-methylethyl)-, (R)- | 95 |
| 29.278 | 9.37 | D-Limonene | 99 |
| 29.521 | 5.47 | Benzene, 1-methyl-3-(1-methylethyl)- | 95 |
|  |  | Cymene | 95 |
| 29.799 | 0.84 | Benzene, 1,2,3-trimethyl- | 95 |
|  |  | Benzene, 1,2,4-trimethyl- | 94 |
| 32.513 | 1.2 | (+)-4-Carene | 95 |
|  |  | Cyclohexene, 1-methyl-4-(1-methylethylidene)- | 95 |
|  |  | 1,3-Cyclohexadiene, 1-methyl-4-(1-methylethyl)- | 95 |
| 33.368 | 1.13 | Benzene, 1-methyl-4-(1-methylethenyl)- | 96 |
|  |  | o-Isopropenyltoluene | 95 |
| 42.027 | 1.2 | Benzothiazole | 95 |
| 48.047 | 0.86 | 1,2,3-Trimethylindene | 70 |
|  |  | 1H-Indene, 1,1,3-trimethyl- | 64 |
| 50.776 | 1.35 | Naphthalene, 2,6-dimethyl- | 97 |
|  |  | Naphthalene, 1,6-dimethyl- | 96 |
|  |  | Naphthalene, 2,7-dimethyl- | 95 |
| 55.282 | 1.11 | Naphthalene, 1,6,7-trimethyl- | 98 |
|  |  | Naphthalene, 1,4,6-trimethyl- | 97 |
|  |  | Naphthalene, 2,3,6-trimethyl- | 97 |
| 59.203 | 0.87 | Heptadecane | 98 |
|  | 57.67 | Others |  |
